# Epizootiological observations on canine microfilaremia in Gujarat state, India

**DOI:** 10.14202/vetworld.2018.1564-1568

**Published:** 2018-11-09

**Authors:** J. R. Patel, S. Devi, J. P. Varshney, K. M. Jadhav

**Affiliations:** 1 Department of Medicine, College of Veterinary Science and Animal Husbandry, Sardarkrushinagar Dantiwada Agricultural University, Sardarkrushinagar, Gujarat, India; 2 Ex-Principal Scientist, Division of Medicine, ICAR-IVRI, Lane, Ghod Dod Road, Opposite Children Traffic Training Park, Surat-395 001, Gujarat, India

**Keywords:** *Dipetalonema reconditum*, *Dirofilaria immitis*, dogs, microfilariae, modified Knott’s technique, prevalence

## Abstract

**Aim:**

The present investigation was conducted to study the prevalence of microfilaremia in dogs in Gujarat.

**Materials and Methods:**

A total of 418 adult dogs aged between 2 and 14 years with signs of weakness and non-specified complaints, presented at TVCC, Deesa (North Gujarat), Nandini Veterinary Hospital, Surat (South Gujarat), and Private Clinics, Ahmedabad (Central Gujarat), were included in the present investigation for studying the prevalence of microfilaremia from July 2016 to May 2017.

**Results:**

A total of 418 dogs were screened, of which 33 were found positive for circulating microfilariae with the prevalence rate of 7.89% in the population. Among microfilaremic dogs, the finding of microfilariae of *Dipetalonema* (*Acanthocheilonema*) reconditum was more common (23 cases; 69.69%) than *Dirofilaria immitis* (10 cases; 30.30%) making their prevalence in the population of 418 dogs as 5.50% and 2.39%, respectively. Breed-wise distribution of microfilaremic dogs revealed that 12 (36.36%), 8 (24.24%), 5 (15.15%), 4 (12.12%), 2 (6.06%), 1 (3.03%), and 1 (3.03%) cases were observed in Pomeranian, non-descript, German Shepherd, Labrador, Great Dane, Lhasa Apso, and Pug dogs, respectively. Of 10 cases of *D. immitis*, 5, 2, 2, and 1 were observed in Pomeranian, Labrador, non-descript, and Great Dane dogs, respectively. Cases of *Dipetalonema reconditum* were highest in Pomeranian (7), followed by non-descript (6), German Shepherd (5), Labrador (2), Great Dane (1), Lhasa Apso (1), and Pug (1). Age-wise distribution recorded significantly (p≤0.01) higher number of cases in adult dogs (4-14 years) for *D. immitis* (30.30%) and *D. reconditum* (39.39%). Sex-wise distribution of microfilaremic dogs showed that male (22/33, 66.66%) was more predisposed to microfilaremia rather than females (11/33, 33.34%). It is apparent from the study that the number of dogs with microfilaremia due to *D. reconditum* was significantly (p≤0.01) higher than that of *D. immitis*.

**Conclusion:**

The present study revealed that microfilaremia due to *D. immitis* and *D. reconditum* is prevalent in the state of Gujarat. The infection with *D. immitis* was associated with severe lung and cardiac pathological manifestations.

## Introduction

Canine vector-borne diseases are an emerging problem worldwide attributable to their frequency of occurrence, morbidity rate, and also to their zoonotic relevance [1]. Major vector-borne pathogens infecting dogs are filarial nematodes, *Ehrlichia canis* (*Rickettsial* bacteria), *Borrelia burgdorferi* (spirochete bacteria), *Anaplasma phagocytophilum* and *Anaplasma platys* (*Rickettsial* bacteria), and *Leishmania infantum* (Protozoa) [2]. The most commonly reported filarial worm in dogs is *Dirofilaria immitis*, distributed in tropical, subtropical, and temperate regions of the world, leading to heartworm disease [3-5]. *Dipetalonema reconditum* causes asymptomatic infection in dogs, but the presence of it in higher numbers leads to the formation of subcutaneous nodules [6]. *D. reconditum* though considered non-pathogenic[4] but is easily confused morphologically with pathogenic *D. immitis*, thus ruling out its occurrence helps in avoiding irrational adulticide therapy. Distinguished morphological characteristics of microfilariae of *D. immitis* are 290-330 µm in length with a straight sharp tail end, whereas *D. reconditum is* 260-283 µm in length, shorter than *D. immitis* with broad caudal end and a curved hook-like tail [7].

In India, the prevalence of *D. immitis* in dogs has been reported from various regions of Assam and Kashmir [8,9]. Dogs are the natural hosts for heartworm, although infection can also be seen in wild canids, domestic and wild felids, as well as human beings [10]. The ubiquitous presence of one or more species of vector competent mosquitoes makes transmission easy and eradication difficult as well as sometimes impossible once a reservoir of microfilaremic canids is established. Different species of mosquitoes of genera *Culex*, *Aedes*, *Anopheles*, *Ochlerotatus*, and *Mansonia* are responsible for the transmission of *D. immitis* [11]. Transmission of infection to other dogs occurs through the bite of mosquito carrying infective L_3_ stage. Prepatent period of *Dirofilaria* spp. microfilariae is about 6-7 months and they mature mainly in the pulmonary arteries and the right ventricle of affected dogs. Clinically, dogs suffering from heartworm disease show cough, dyspnea, weight loss, exercise intolerance, weakness, hemoptysis, cyanosis, and congestive heart failure as the main findings [12,13]. The degree of cardiac damage and other clinical manifestations depends on worm burden, its establishment at the predilection site and hosts immune response.

In Gujarat state, India, little information is available regarding the prevalence of canine microfilaremia. Hence, the goal of the present study was to record the prevalence of microfilariae in canines of Gujarat state.

## Materials and Methods

### Ethical approval

The present study was based on clinical cases; hence approval from Institutional Animal Ethics Committee is not required. However, samples were collected as per standard collections procedure without any harm to the animals.

### Animals

A total of 418 dogs aged between 2 and 14 years with signs of weakness and non-specified complaints, presented at TVCC, Deesa (North Gujarat), Nandini Veterinary Hospital, Surat (South Gujarat), and private clinics, Ahmedabad (Central Gujarat), from July 2016 to May 2017, were included in the present investigation. From all the dogs, 1.5 ml of whole blood from the saphenous vein in ethylenediaminetetraacetic acid vial was collected at the time of presentation at the respective clinics for clinical investigation. Modified Knott’s Technique (MKT) as per standard procedures was used to detect circulating microfilariae [14]. The differentiation of *D. immitis* and *D. reconditum* was mainly based on morphological characteristics of microfilariae [7].

### MKT

Using a 15 ml centrifuge tube, approximately 9 ml of 2% formalin was added to 1 ml of anticoagulated blood. The tube was inverted several times to mix it thoroughly and centrifuged for 10 min at 1500 rpm. The supernatant fluid was poured off and 2 drops of 1:1000 aqueous Giemsa stain were mixed to the sediments and shaken well and finally examined under low power objective of a microscope for the presence of microfilariae. The technique was also useful to differentiate between *D. immitis* and *D. reconditum* morphologically on the basis of cephalic hook tail of the parasite.

### Statistical analysis

The prevalence data were subjected to *χ*^2^ test through Statistical Package for the Social Sciences. Version 19 (release 19.0.0, IBM, USA) software.

## Results

In the present study, 418 pet dogs were included and tested for circulating microfilaria and 33 (7.89%) were found positives for the same application of MKT. Among the 33 positives, 10 were positive to *D. immitis* and 23 to *D. reconditum*. The prevalence of *D. immitis* and *D. reconditum* in the population of 418 dogs was recorded as 2.39% and 5.50% (Table-1). Although known to us the lower pathogenicity of *D. reconditum* as compared to other filarioids, its occurrence is screened to differentiate it from pathogenic *D. immitis*. Among the various breeds presented with the associated complaints, the prevalence was recorded to be highest in Pomeranian (15.15% and 21.21%) followed by non-descript (6.06% and 18.18%) breed of dogs for *D. immitis* and *D. reconditum* group, respectively (Table-2). Age-wise prevalence of *D. immitis* and *D. reconditum* was found significantly higher in adult dogs (4-14 years) by Chi-square analysis (p≤0.01) (Table-3). The prevalence of *D. immitis* and *D. reconditum* among the positive population was recorded highest in males (18.80% and 48.48%) than females (12.12% and 21.21%), respectively (Table-4).

**Table-1:** Overall prevalence and distribution of microfilariae (*D. immitis* and *D. reconditum*) in dogs from Gujarat state, India.

Details	Microfilaria (n=33)		Total screened population (n=418)

	*Dirofilaria immitis*	*Dipetalonema reconditum*	
No.	10	23	33
Prevalence % (disease)	30.30	69.69	7.89

**Table-2:** Overall prevalence and distribution of microfilariae (*D. immitis* and *D. reconditum*) in different breeds of dogs from Gujarat state, India.

Breed	Details	Microfilaria (n=33)	

		*Dirofilaria immitis* (n=10)	*Dipetalonema reconditum* (n=23)
P*	n	5	7
	Prevalence % (disease)	50	30.43
	% of total	15.15	21.21
L*	n	2	2
	Prevalence % (disease)	20	8.69
	% of total	6.06	6.06
ND*	n	2	6
	Prevalence % (disease)	20	26.08
	% of total	6.06	18.18
GD*	n	1	1
	Prevalence % (disease)	10	4.34
	% of total	3.03	3.03
GS*	n	00	5
	Prevalence % (disease)	00	21.73
	% of total	00	15.15
LA*	n	00	1
	Prevalence % (disease)	00	4.34
	% of total	00	3.03
PG*	n	00	1
	Prevalence % (disease)	00	4.34
	% of total	00	3.03

P*=Pomeranian, L*=Labrador, ND*=Non-descript, GD*=Great Dane, GS*=German Shepherd, LA*=Lhasa Apso, PG*=Pug

**Table-3:** Overall prevalence and distribution of microfilariae (*D. immitis* and *D. reconditum*) in different age groups of dogs from Gujarat state, India.

Age group	Details	Microfilaria (n=33)		Total (n=33)

		*Dirofilaria immitis* (n=10)	*Dipetalonema reconditum* (n=23)	
2-4 years	n	0	10	10
	Prevalence % (disease)	0	43.47	
	% of total	0	30.30	
4-14 years	n	10*	13	23
	Prevalence % (disease)	100	56.52	
	% of total	30.30	39.39	

*χ*^2^ - value, df=6.238, 1; P=0.01

**Table-4:** Overall prevalence and distribution of microfilariae (*D. immitis* and *D. reconditum*) in male and female dogs from Gujarat state, India.

Sex	Details	Microfilaria (n=33)	

		*Dirofilaria immitis* (n=10)	*Dipetalonema reconditum* (n=23)
Male	n	6	16
	Prevalence % (disease)	60	69.57
	% of total	18.18	48.48
Female	n	4	7
	Prevalence % (disease)	40	30.43
	% of total	12.12	21.21

## Discussion

Filariasis caused by several species of filarids is a silent killer disease among canine population. *D. immitis*, the most pathogenic canine filarid, is responsible for heartworm disease in dogs. In the present study, the prevalence of *D. immitis* and *D. reconditum* in suspected dogs was recorded as 30.30% (10/33) and 69.69% (23/33), whereas prevalence in the population of 418 dogs as 2.39% and 5.42%, respectively. Bhattacharjee and Sarmah [15] recorded the prevalence rate of 5.42% of *D. immitis* in a study of 424 clinically ill dogs from Assam on the basis of wet film examination. The most common species of microfilariae identified in the present study was *D. reconditum* followed by *D. immitis*. Similar finding reported in a study [6] on total population of 525 dogs. The increase in the recognition and diagnosis of filarial nematodes might be due to the ubiquitous presence of intermediate hosts (fleas, lice, ticks, mosquitoes, etc.) among dog population at any time and place as an impact of climate change, spectacular advancement in the availability of techniques/methods in the diagnosis of diseases, increased awareness of pet owners toward the health of their dogs, and most importantly lack of proper prophylactic schedule.

Pomeranian and non-descript breeds of dog showed increased propensity toward microfilaremia. Lefkaditis *et al*. [16] reported 15 (1.68%) and 10 (1.12%) cases of *D. immitis* in mix and pure breed dogs from a total population of 893 dogs screened in Greece. The exact cause of these findings is not explainable, since, in any geographical area, the breed incidence may be affected by the preference of specific breeds to be kept by the owners of that area according to work explored with them. Furthermore, importantly due to a smaller number of cases relative to particular breeds, any further inference could not be substantiated.

In our study, statistically higher prevalence of microfilaremia was observed in adult dogs (4-14 years) supported by the findings of Simon *et al*. [17] who reported the higher prevalence rate of *D. immitis* in older dogs. Oge *et al*. [18] concluded that increased prevalence of *D. immitis* in older dogs is due to lack of the age resistance factor and longer exposure to the mosquitoes. It may be suggested that the movement of dogs and the increased activity during this age group may be the possible reasons for harboring these problems in adult dogs [19]. On the contrary, younger age group reported no case of heartworm and a lesser number of *D. reconditum* cases in the present study. Our findings are in close association with Labarthe *et al*. [20] who found that younger dogs (1-2 year) were less likely to be detected positive than dogs of older age groups. Long prepatent period (6-7 months) needed to complete the life cycle of filarial worms in dogs was also proposed as the reason for the same.

Sex-wise prevalence of *D. immitis* and *D. reconditum* among the positive population was recorded to be highest in males. Bohloli *et al*. [21] and Liu *et al*. [22] opined that male dogs are more commonly affected with *D. immitis* rather than females. It might be due to the more preference of male dogs as a pet by the owners for guarding which, in turn, will expose them to external environment for a longer period of time, thus to the vectors [23]. Male and large-sized dogs are more likely to be infected by *Dirofilaria* spp., possibly because animals living outdoor and of large size are more exposed to mosquito bites and also for longer period [24].

In conclusion, canine filariasis is prevalent in the state of Gujarat, and pattern of prevalence appears to be influenced mainly by the vector population, environment, and lifestyle of pets as well as of the owners. *D. reconditum* should be screened to differentiate it from *D. immitis*. Canines should always be screened for the presence of microfilaria to prevent mortality and zoonotic transmission. The prevalence of microfilariae is relevant to both human and veterinary public health, contributing to the general awareness of pet owners and veterinarian practitioners and reinforcing the need for effective control measures against vectors and preventive therapy in companion animals.

## Authors’ Contributions

JRP did the study as the postgraduate research student. SD and JPV guided, designed the research work, and drafted the manuscript. KMJ corrected the manuscript. All authors read and approved the final manuscript.
